# Trional and Tetronal

**Published:** 1894-02-10

**Authors:** 


### TRIONAL AND TETRONAL.

Trional and tetronal are interesting new drugs, for they are substitution derivatives from sulphonal, which they very closely resemble in their action. In trional one of the methyl groups of sulphonal is replaced by an ethyl group, and in tetronal two of the methyl groups are replaced by ethyl groups, so that tetronal is di-ethyl-sulpho-dimethylmetane. Trional consists[1] of brilliant crystals, soluble 1 in 300 of cold water, more soluble in warm water or alcohol. It has a slightly bitter taste. Tetronal is also a crystalline substance, soluble 1 in 400 of cold water, and much more soluble in warm water or alcohol; it tastes like trional, and has a camphoraceous odour. Physiological experiments[2] have shown that these drugs are powerful depressants of the central nervous system; first the animal becomes drowsy, and later on there is paralysis from depression of the functions of the spinal cord, and finally death takes place. The blood, the circulation, the respiration, and the temperature are unaltered. Tetronal is slightly the more powerful of the two drugs. Occasionally a cumulative action has been noticed. The best way to obtain the physiological effects of these drugs is to administer them either suspended or dissolved in alcohol. Both have had an extended trial on the human subject, especially in Russia[3], and many doctors have taken them themselves. The action of both is the same, namely, hypnotic. Trional has been most used, and a dose of fifteen to thirty grains quickly produces drowsiness, followed by calm, refreshing sleep, from which the patients wake much refreshed, and usually without any ill-effects, although occasionally there is a slight headache, which quickly passes off. Women and children require smaller doses than those just mentioned. Combined with opium it has been found useful for melancholia, and by itself it has been given with great benefit in mania. No cases of trional habit are on record. Unpleasant symptoms are much rarer than after sulphonal. They are depression of motor and mental power, inco-ordination of movement, headache, and cardiac depression. The dose of tetronal is the same as that of trional, and should be given for the same cases. At present no difference has been detected in the action of these two drugs. In Russia both are cheap. They are usually given in some warm vehicle half an hour before going to bed.

[1] Raimodi et Mariottini, Nouv. Rev., 1892, p. 505. [2] Ibid. [3] Randa Vratch, 1893, No. 9.

